# Impact of Foliar Application of Amino Acids on Total Phenols, Phenolic Acids Content of Different Mints Varieties under the Field Condition

**DOI:** 10.3390/plants10030599

**Published:** 2021-03-23

**Authors:** Živilė Tarasevičienė, Aloyzas Velička, Aurelija Paulauskienė

**Affiliations:** Faculty of Agronomy, Institute of Agricultural and Food Sciences, Vytautas Magnus University Agriculture Academy, Donelaicio str. 58, 44248 Kaunas, Lithuania; aloyzas.velicka@vdu.lt (A.V.); aurelija.paulauskiene@vdu.lt (A.P.)

**Keywords:** aromatic amino acids, *Mentha spicata*, *Mentha piperita*, phenolic acids

## Abstract

Phenolic compounds have a number of benefits to human health and can be used as preventive compounds for the development of some chronic diseases. *Mentha* plants are not only a good source of essential oils, but also contain significant levels of wide range of phenolic compounds. The aim of this research was to investigate the possibility to increase phenols content in *Mentha* plants under the foliar application with L-phenylalanine, L-tryptophan, L-tyrosine at two concentrations (100 mg L^−1^ and 200 mg L^−1^) and to create preconditions for using this plant for even more diverse purposes. Quantitative and qualitative analyses of phenols in mints were performed by HPLC method. Foliar application of amino acids increased the total phenol content from 1.22 to 3.51 times depending on the treatment and mint variety. The most pronounced foliar application to total phenols content was tryptophane especially in *Mentha piperita* “Swiss”. *Mentha piperita* “Swiss” was affected most by foliar application and the amount of total phenolic acids depending on the treatment ranged from 159.25 to 664.03 mg 100 g^−1^ (DW), respectively, non-sprayed and sprayed with tryptophane 100 mg L^−1^. Our results suggest that the biophenol content varies according to such factors as foliar application and variety, and every single mint variety has individual response to different applications of amino acids.

## 1. Introduction

Mint plants (*Mentha* spp.) are among the world’s oldest and most popular herbs. The plants of this family are widely distributed in moderate temperature regions in all continents except Antarctica. The most economically important mint species are *M. aquatica* L., *M. canadensis* L., *M. spicata* L. and three other their hybrids, the most known related to *M. piperita* L. (a sterile hybrid between *M. aquatica* and *M. spicata*) [1]. Mint essential oil is one of the most important mint products that is widely used for medical purposes, in the food and beverage industry, for relaxation, and also as fragrances in various industries. Mint essential oils and extracts are used to treat fever, cough and digestive disorders [2]. Due to the presence of phenolic compounds, phytosterols and unsaturated fatty acids, some species of mints have antioxidant and anti-cancer effect [3,4,5]. Hanafy et al.’s [6] findings suggest that mints provide a substantial basis for future research into Alzheimer’s disease treatment because of the total phenolic content, antioxidant capacity, acetylcholinesterase (AChE) and histone deacetylase (HDAC) inhibition activities. Atanassova et al. [7] found 45.25 mg GAE 100 g^−1^ DW total phenolic content and 25.17 mg CE 100 g^−1^ DW total flavonoid content in *M. piperita*. According to Hanafy et al. [6], total phenolic content in mints belonging to section *Mentha* ranged from 2.82 mg to 8 mg GAE g^−1^ DW. Luteolin, naringenin, apigenin, hesperitin and eriodictyol and their glycosides, as well as rosmarinic, caffeic, chlorogenic and salvianolic acids are the main phenolic compounds in mints plants [8]. According to Kivilompolo et al. [9] the main phenolic acids in *M. spicata* plants grown in Finland were gallic, chlorogenic, caffeic, vanillic, syringic, p-coumaric, ferulic, and rosmarinic acids, while *M. piperita* plants from Croatia included rosmarinic, caffeic, gallic, syringic, p-hydroxybenzoic, o-coumaric, and cinnamic acids [10]. The quality and quantity of biologically active substances in plants depend on genotype, soil, climatic conditions, fertilisation and harvest time, as well as on abiotic and biotic stress [11]. The activation of the synthesis of biologically active compounds can be achieved using various biostimulants, including amino acids. Recent studies have shown that the use of biostimulants with amino acids in agriculture can increase the yield, metabolism and the biosynthesis of plant metabolites in tea, lemon balm, basil, and rosemary [12,13,14,15,16]. The aromatic amino acids L-phenylalanine, L-tyrosine, and L-tryptophan are not only components important for protein synthesis, but they can also be used as precursors to increase secondary metabolite content in plants. These amino acids play an important role in the shikimate pathway and also many compounds, which are derived from them: alkaloids, indole glucosinolates, flavonoids, hydroxycinnamic acids, lignins, and lignans [17,18]. The principal aromatic phenolic compounds synthesized from phenylalanine and tyrosine are cinnamic acids and esters, coumarins, phenylpropenes, chromones, stilbenes, anthraquinones, chalcones, flavonoids, isoflavonoids, neoflavonoids, and their dimers and trimers, respectively, lignans, neolignans, aromatic polyketides, and diphenylheptanoids [19]. Ardebili et al. [20] determined that foliar spraying with amino acids significantly induced phenylalanine ammonia lyase (PAL) activities and improved phenol content in *Aloe vera* L plants. Watts et al. [21] reported that L-tyrosine ammonia-lyase was also involved in the synthesis of phenolic compounds via the shikimic pathway. Musbah and Ibrahim [22] determined that tyrosine and phenylalanine at the concentrations 0.20, 0.25 and 0.30 g L^−1^ added either by spraying on the vegetative parts or supplemented to the tissue culture medium showed positive influence on the number of total phenols in *Coleus blumei* plants. L-tryptophan is a precursor in plant hormone auxin synthesis [23]. Phenolic compounds influence different physiological processes related to growth and development in plants: seed germination and cell division, as well as the synthesis of photosynthetic pigments, growth bioregulators [24,25,26]. Plants exhibiting increased synthesis of polyphenols under abiotic stresses in most of the cases show a better adaptability [26].

Considering the above, the results from other researchers suggest that phenolic compounds are very important for plants physiological processes and adaptability as well as for human health. Mints as an important industrial plant are used in many industries, especially in pharmacy and food where, with increased phenols content, they can be used even more widely. Therefore, the aim of this research was to investigate *Mentha* plants’ response to the foliar application of different aromatic amino acids under the field conditions and increase the bioactive phenols content.

## 2. Results

### 2.1. Total Phenol Content

**Figure 1:**
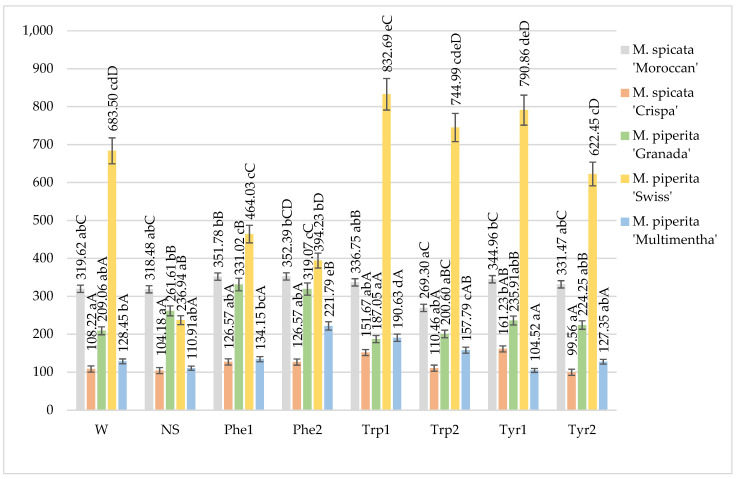
Total phenols content in mints influenced by foliar application of amino acids, mg 100 g^−1^ DM in 2017–2018 (W—water, NS—non sprayed, Phe1—phenylalanine 100 mg L^−1^, Phe2—phenylalanine 200 mg L^−1^, Trp1—tryptophane 100 mg L^−1^, Trp2—tryptophane 200 mg L^−1^, Tyr1—tyrosine 100 mg L^−1^, Tyr2—tyrosine 200 mg L^−1^; Means marked with different upper letters (A, B, C…) indicate significant difference between varieties at *p* < 0.05; Means marked with different lower letters (a, b, c…) indicate significant difference between foliar application with amino acids at *p* < 0.05).

The total phenol content in the mints fluctuated from 99.56 in *M. spicata* “Crispa” to 832.69 mg 100 g^−1^ in *M. piperita* “Swiss”. The highest total phenol content in the non-sprayed mints was observed in *M. spicata* “Moroccan” while the lowest one was found in *M. spicata* “Crispa” and *M. piperita* “Multimentha” (Figure 1).

The water application was the most effective for *M. piperita* “Swiss” as well as phenylalanine at different concentrations also was the most effective in *M. piperita* “Swiss”. An increase in the total phenol content in *M. piperita* “Swiss” was achieved by the application of tryptophan 100 mg L^−1^, 200 mg L^−1^ and tyrosine 100 mg L^−1^. Only the application of tryptophan 100 mg L^−1^ significantly increased total phenols comparing with the water sprayed mints. Foliar applied solutions differently influenced the amount of total phenols in the mints. The foliar application of phenylalanine at concentrations of 100 and 200 mg L^−1^ significantly increased the amount of total phenols in *M. piperita* “Granada” compared with otherwise treated plants. Phenylalanine at a concentration of 200 mg L^−1^ also showed a positive effect on total phenols in *M. piperita* “Multimentha” while tyrosine 100 mg L^−1^—on *M. spicata* “Crispa”. The highest amount of total phenols accumulated in *M. piperita* “Swiss” under the foliar application with tryptophan and tyrosine at different concentrations, compared with other mint varieties (Figure 1).

### 2.2. Total Phenolic Acids Content

**Figure 2:**
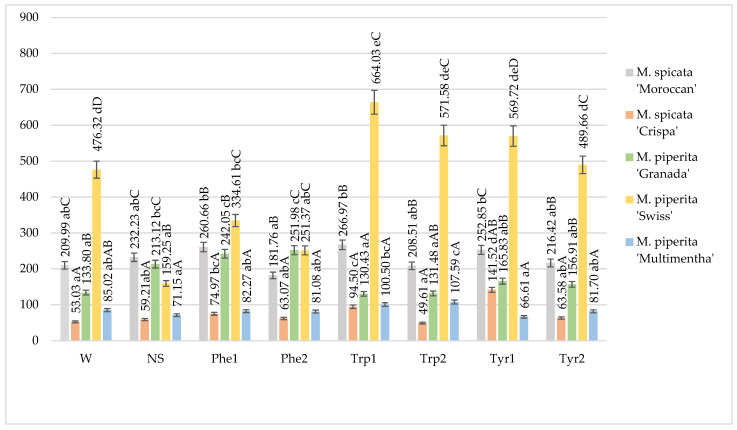
Total phenolic acids content in mints influenced by foliar application of amino acids, mg 100 g^−1^ DM in 2017–2018 (W—water, NS—non sprayed, Phe1—phenylalanine 100 mg L^−1^, Phe2—phenylalanine 200 mg L^−1^, Trp1—tryptophane 100 mg L^−1^, Trp2—tryptophane 200 mg L^−1^, Tyr1—tyrosine 100 mg L^−1^, Tyr2—tyrosine 200 mg L^−1^; Means marked with different upper letters (A, B, C…) indicate significant difference between varieties at *p* < 0.05; Means marked with different lower letters (a, b, c…) indicate significant difference between foliar application with amino acids at *p* < 0.05).

It was observed that the amount of phenolic acids was influenced by mint variety as well as foliar application of amino acids (Figure 2).

The highest amount of phenolic acids was determined in *M. piperita* “Swiss” and depending on the application of amino acids ranged from 159.25 to 664.03 mg 100 g^−1^ (DW). The application with tryptophan 100 mg L^−1^ significantly influenced the highest amount of phenolic acids in *M. piperita* “Swiss” compared with other treatments and mints without foliar application and sprayed with water. A statistically significant difference between the concentrations of amino acids for *M. piperita* “Swiss” was not observed. The least amount of phenolic acids was observed in *M. spicata* “Crispa” and the foliar application with tyrosine 100 mg L^−1^ significantly influenced the highest amount of phenolic acids, compared with other treatments. The lowest concentrations of amino acids statistically significantly influenced the higher amount of phenolic acids than at higher concentrations. Foliar spray application of tryptophan solutions had a significant impact on phenolic acid content in *M. piperita* “Multimentha” compared with other treated mints. In *M. piperita* “Granada”, only the application of phenylalanine had a positive impact on the amount of phenolic acids while in *M. spicata* “Moroccan”, foliar-applied solutions had no influence on the accumulation of bioactive compounds (Figure 2).

Irrespective of foliar application, the highest amounts of phenolic acids were determined in *M. piperita* “Swiss” and the lowest ones—in *M. spicata* “Crispa” and *M. piperita* “Multimentha” (Figure 2).

### 2.3. Hydroxybenzoic Acids Content

A predominant hydroxybenzoic acid in mints was gallic acid, the amount of which fluctuated from 4.11 to 24.81 mg 100 g^−1^, while benzoic acid fluctuated from 0.53 to 8.90 mg 100 g^−1^ (Table 1 and Table 2). The application of aromatic amino acids did not influence the gallic acid content in *M. spicata* “Moroccan”, while the amount of benzoic acid after the application of amino acids solution and water decreased compared with non-sprayed mints. Phenylalanine at different concentrations statistically significantly influenced the amount of gallic acid in *M. spicata* “Crispa” and *M. piperita* “Granada” as well as phenylalanine 200 mg L^−1^ influenced the amount of benzoic acid in *M. spicata* “Crispa” and *M. piperita* “Swiss”. After foliar application, the amount of gallic acid in *M. piperita* “Swiss” decreased with the exception of the tryptophane solutions tested (Table 1). The same tendencies were observed in *M. piperita* “Multimentha” and only the solution of tryptophane 100 mg L^−1^ influenced the significant increase in gallic acid as well as in benzoic acid (Table 1 and Table 2). 

The influence of the tested solutions of tryptophane and tyrosine at 100 mg L^−1^ concentration was more pronounced for benzoic acid in *M. piperita* “Multimentha”. The water application statistically significantly influenced the highest amount of benzoic acid only in *M. piperita* “Granada” (Table 2). Independent of the foliar application, the highest amount of gallic acid was observed in *M. piperita* “Swiss” and *M. piperita* “Multimentha”, with, respectively, 24.81 and 16.70 mg 100 g^−1^. After assessing the influence of the variety on the accumulation of hydroxybenzoic acids, it was established that the highest amounts of those acids regardless of the foliar application was present in *M. piperita* “Swiss” and *M. piperita* “Multimentha” (Table 1 and Table 2).

### 2.4. Hydroxycinnamic Acids Content

The research data show that the variety of mints, as well as foliar application, have a significant influence on the phenolic acid content in plants (Table 3, Table 4, Table 5, Table 6 and Table 7). The predominant hydroxycinnamic acid in non-sprayed mints was *p*-coumaric acid and its amount ranged from 30.16 in *M. piperita* “Multimentha” to 117.27 mg 100 g^−1^ in *M. spicata* “Moroccan” (Table 5). Foliar application of amino acids did not affect the amount of chlorogenic acid in *M. spicata* “Moroccan” while the highest amount in *M. spicata* “Crispa” was observed after the treatment with tyrosine 200 mg L^−1^. Tyrosine solution with 100 mg L^−1^ of active ingredients was more pronounced in *M. piperita* “Granada” and amount of chlorogenic acid after its application was 19.42 mg 100 g^−1^. All applied treatments in *M. piperita* “Swiss” were effective for chlorogenic acid accumulation compared with the non-sprayed plants while phenylalanine 200 mg L^−1^ was effective in *M. piperita* “Multimentha” (Table 3).

Caffeic acid in the mints ranged from 5.18 in *M. piperita* “Multimentha” to 340.87 mg 100 g^−1^ in *M. piperita* “Swiss” (Table 4). While comparing amounts of caffeic acid in the non-sprayed mints, the highest amount was detected in *M. spicata* “Moroccan” and the lowest one—in *M. spicata* “Crispa” and *M. piperita* “Multimentha”; respectively, 55.93 and 7.64, 7.50 mg 100 g^−1^. The application of tyrosine 100 mg L^−1^ increased the amount of caffeic acid in *M. spicata* “Crispa”, while foliar application had no statistically significant influence on *M. spicata* “Moroccan”. Tryptophane at different concentrations decreased the amount of caffeic acid in *M. piperita* “Granada”, while in *M. piperita* “Swiss”, it was increased by 15.09 times compared with the non-sprayed mints (Table 4).

The amount of *p*-coumaric acid in mints fluctuated from 15.14 in *M. piperita* “Swiss” to 134.99 mg 100 g^−1^ in *M. piperita* “Granada” (Table 5). The highest amount of *p*-coumaric acid in the non-sprayed mints was found in *M. spicata* “Moroccan” and *M. piperita* “Granada”, respectively, 117.27 and 105.84 mg 100 g^−1^. All foliar treatments had no effect on the amount of *p*-coumaric acid in *M. spicata* “Moroccan”, while phenylalanine 200 mg L^−1^ decreased. The opposite effect was observed in *M. piperita* “Granada” after the application of phenylalanine where the amount *p*-coumaric acid did not decrease but was the same as in the non-sprayed mints. Foliar application of tryptophane had the same influence on *p*-coumaric acid accumulation in *M. piperita* “Granada”, *M. piperita* “Swiss”, *M. piperita* “Multimentha” as water (Table 5).

The highest content of ferulic acid was determined in *M. piperita* “Swiss” as well as that of chlorogenic acid (Table 3 and Table 6). Foliar application of water increased that acid content in *M. piperita* “Swiss” by 7.73 times compared with the non-sprayed plants. Variety characteristics had significant influence on ferulic acid content in the mints as well as the treatment. The highest amount of ferulic acid in the non-sprayed mints was 27.10 in *M. piperita* “Swiss” and the lowest was 3.16 mg 100 g^−1^ in *M. spicata* “Crispa”. Different foliar applications influenced significant increase in ferulic acid in different mints compared with the non-sprayed plants: tyrosine 200 mg L^−1^ in *M. spicata* “Crispa”, as well as tyrosine 100 mg L^−1^ in *M. piperita* “Granada” and phenylalanine 100 mg L^−1^ in *M. piperita* “Multimentha” (Table 6).

The cinnamic acid content in the mints ranged from 0.07 in *M. spicata* “Crispa” and *M. piperita* “Multimentha” to 23.40 mg 100 g^−1^ in *M. piperita* “Granada” (Table 7). The most effective foliar application solution was tyrosine 100 mg L^−1^ after the application of which the cinnamic acid content in *M. spicata* “Crispa” increased 33 times, and it increased 19.48 times in *M. piperita* “Swiss” compared with the non-sprayed mints. Research data show that phenylalanine at different concentrations had statistically significant negative influence on the cinnamic acid content in *M. spicata* “Moroccan” and *M. piperita* “Granada” (Table 7).

### 2.5. Principal Component (PCA) and Hierarchical Clustering Analysis (HCA)

**Figure 3:**
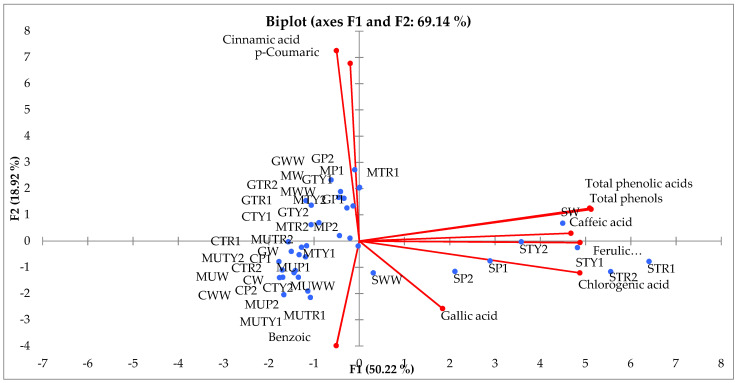
Principal-component analysis (PCA) for total phenolic, total phenolic acids content, amount of phenolics acids of different varieties of mints influenced by different amino acids and its concentrations (MW—*M. spicata* “Moroccan” water, MWW—*M. spicata* “Moroccan” non-sprayed, MP1—*M. spicata* “Moroccan” phenylalanine 100 mg L^−1^, MP2—*M. spicata* “Moroccan” phenylalanine 200 mg L^−1^, MTR1—*M. spicata* “Moroccan” tryptophan 100 mg L^−1^, MTR2—*M. spicata* “Moroccan” tryptophan 200 mg L^−1^, MTY1—*M. spicata* “Moroccan” tyrosine 100 mg L^−1^, MTY2—*M. spicata* “Moroccan” tyrosine 200 mg L^−1^, CW—*M. spicata* “Crispa” water, CWW—*M. spicata* “Crispa” non-sprayed, CP1—*M. spicata* “Crispa” phenylalanine 100 mg L^−1^, CP2—*M. spicata* “Crispa” phenylalanine 200 mg L^−1^, CTR1—*M. spicata* “Crispa” tryptophan 100 mg L^−1^, CTR2—*M. spicata* “Crispa” tryptophan 200 mg L^−1^, CTY1—*M. spicata* “Crispa” tyrosine 100 mg L^−1^, CTY2—*M. spicata* “Crispa” tyrosine 200 mg L^−1^, GW—*M. piperita* “Granada” water, GWW—*M. piperita* “Granada” non-sprayed, GP1—*M. piperita* “Granada” phenylalanine 100 mg L^−1^, GP2—*M. piperita* “Granada” phenylalanine 200 mg L^−1^, GTR1—*M. piperita* “Granada” tryptophan 100 mg L^−1^, GTR2—*M. piperita* “Granada” tryptophan 200 mg L^−1^, GTY1—*M. piperita* “Granada” tyrosine 100 mg L^−1^, GTY2—*M. piperita* “Granada” tyrosine 200 mg L^−1^, SW—*M. piperita* “Swiss” water, SWW—*M. piperita* “Swiss” non-sprayed, SP1—*M. piperita* “Swiss” phenylalanine 100 mg L^−1^, SP2—*M. piperita* “Swiss” phenylalanine 200 mg L^−1^, STR1—*M. piperita* “Swiss” tryptophan 100 mg L^−1^, STR2—*M. piperita* “Swiss” tryptophan 200 mg L^−1^, STY1—*M. piperita* “Swiss” tyrosine 100 mg L^−1^, STY2—*M. piperita* “Swiss” tyrosine 200 mg L^−1^, MUW—*M. piperita* “Multimentha” water, MUWW—*M. piperita* “Multimentha” non-sprayed, MUP1—*M. piperita* “Multimentha” phenylalanine 100 mg L^−1^, MUP2—*M. piperita* “Multimentha” phenylalanine 200 mg L^−1^, MUTR1—*M. piperita* “Multimentha” tryptophan 100 mg L^−1^, MUTR2—*M. piperita* “Multimentha” tryptophan 200 mg L^−1^, MUTY1—*M. piperita* “Multimentha” tyrosine 100 mg L^−1^, MUTY2—*M. piperita* “Multimentha” tyrosine 100 mg L^−1^).

The principal component analysis (PCA) was performed to evaluate the relationships between the applications of the amino acids, total amounts of phenols, phenolic acids and their compositions during the period 2017–2018. The first two components (PCs) were associated with eigenvalues higher than one and explained 50.22% and 18.22% of total variance (Figure 3). The first factor (PC1) was highly and positively correlated with total phenols, total phenolic acids, caffeic, gallic, ferulic and chlorogenic acids while the second factor (PC2) highly and positively related with *p*-coumaric and cinnamic acids and negatively with benzoic ones (Figure 3).

Figure 3 shows that all phenolic acids, as well as the total amount of phenols and the total amount of phenolic acids, differed with different application of amino acids, whereas all treatments and varieties of mints were well separated in the PCA map. The principal component analysis shows that the highest content of total phenols, total phenolic acid content, the amount of caffeic, gallic, ferulic and chlorogenic acids were associated with all amino acids used in the experiment and foliar applied on the *M. piperita* “Swiss”. Other amino acid treatments were more effective on the *p*-coumaric and cinnamic acid content in other remaining mint varieties. Almost all treatments showed the negative effect of benzoic acid content on all mint varieties. 

**Figure 4:**
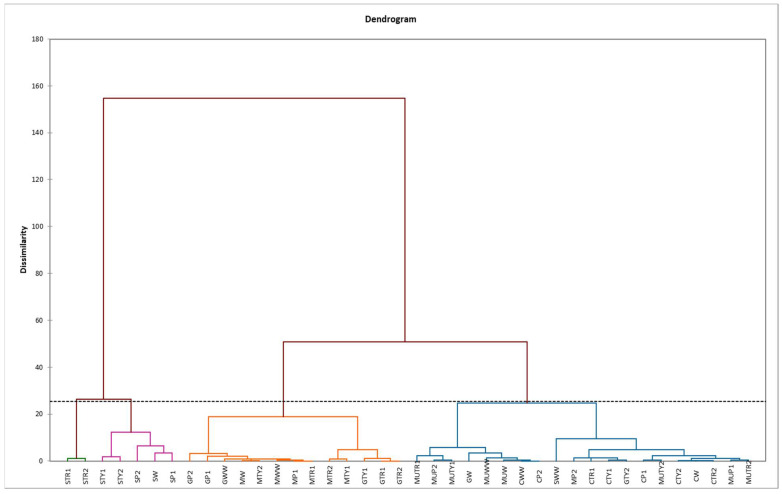
Hierarchical clustering analysis (HCA) of different mints influenced by foliar application of amino acids (MW—*M. spicata* “Moroccan” water, MWW—*M. spicata* “Moroccan” non-sprayed, MP1—*M. spicata* “Moroccan” phenylalanine 100 mg L^−1^, MP2—*M. spicata* “Moroccan” phenylalanine 200 mg L^−1^, MTR1—*M. spicata* “Moroccan” tryptophan 100 mg L^−1^, MTR2—*M. spicata* “Moroccan” tryptophan 200 mg L^−1^, MTY1—*M. spicata* “Moroccan” tyrosine 100 mg L^−1^, MTY2—*M. spicata* “Moroccan” tyrosine 200 mg L^−1^, CW—*M. spicata* “Crispa” water, CWW—*M. spicata* “Crispa” non-sprayed, CP1—*M. spicata* “Crispa” phenylalanine 100 mg L^−1^, CP2—*M. spicata* “Crispa” phenylalanine 200 mg L^−1^, CTR1—*M. spicata* “Crispa” tryptophan 100 mg L^−1^, CTR2—*M. spicata* “Crispa” tryptophan 200 mg L^−1^, CTY1—*M. spicata* “Crispa” tyrosine 100 mg L^−1^, CTY2—*M. spicata* “Crispa” tyrosine 200 mg L^−1^, GW—*M. piperita* “Granada” water, GWW—*M. piperita* “Granada” non-sprayed, GP1—*M. piperita* “Granada” phenylalanine 100 mg L^−1^, GP2—*M. piperita* “Granada” phenylalanine 200 mg L^−1^, GTR1—*M. piperita* “Granada” tryptophan 100 mg L^−1^, GTR2—*M. piperita* “Granada” tryptophan 200 mg L^−1^, GTY1—*M. piperita* “Granada” tyrosine 100 mg L^−1^, GTY2—*M. piperita* “Granada” tyrosine 200 mg L^−1^, SW—*M. piperita* “Swiss” water, SWW—*M. piperita* “Swiss” non-sprayed, SP1—*M. piperita* “Swiss” phenylalanine 100 mg L^−1^, SP2—*M. piperita* “Swiss” phenylalanine 200 mg L^−1^, STR1—*M. piperita* “Swiss” tryptophan 100 mg L^−1^, STR2—*M. piperita* “Swiss” tryptophan 200 mg L^−1^, STY1—*M. piperita* “Swiss” tyrosine 100 mg L^−1^, STY2—*M. piperita* “Swiss” tyrosine 200 mg L^−1^, MUW—*M. piperita* “Multimentha” water, MUWW—*M. piperita* “Multimentha” non-sprayed, MUP1—*M. piperita* “Multimentha” phenylalanine 100 mg L^−1^, MUP2—*M. piperita* “Multimentha” phenylalanine 200 mg L^−1^, MUTR1—*M. piperita* “Multimentha” tryptophan 100 mg L^−1^, MUTR2—*M. piperita* “Multimentha” tryptophan 200 mg L^−1^, MUTY1—*M. piperita* “Multimentha” tyrosine 100 mg L^−1^, MUTY2—*M. piperita* “Multimentha” tyrosine 100 mg L^−1^).

Based on the HCA, mints samples were clustered into four clusters (C1, C2, C3 and C4) (Figure 4). The first cluster (C1) was formed by 13 foliar applied mints with the highest content of p-coumaric and cinnamic acids; the second cluster (C2) by 20 foliar applied mints with the lowest content of all phenols; the third cluster (C3) by five foliar applied mints with intermediate amount of all observed phenols and the fourth cluster (C4) by *M. piperita* “Swiss” foliar applied with tryptophan with the highest amount of all investigated phenols, except cinnamic acid (Figure 4).

## 3. Discussion

Phenolic compounds constitute a chemically heterogenous groups with different properties as well as solubility [27]. Research studies have been performed to investigate the possibilities of stimulation of the synthesis of phenols using aromatic amino acids in vivo and in vitro with different plants, but it is obvious that there is still not enough data to make conclusions how these precursors are effective for the promotion of phenolic compound synthesis in mints under field conditions [28,29,30].

A relatively large variation in the total phenolic content of different mint varieties was recorded in this study. However, this does not contradict the data provided by Hanafy et al. [6] where it is indicated that, depending on mint taxa, the average amount of total phenols is 5.1 mg GAE g^−1^ DW.

The investigated measures showed that stability in both experimental years and a statistically significant interaction between years and amounts of phenolic compounds was not found. It was also established that the variety and treatment, as well as their interaction, were statistically significant (*p* < 0.05).

It was revealed that, depending on the variety, the treatment of all used amino acids influenced the increase in the total number of phenolic compounds compared with the non-treated plants. The efficiency of tyrosine 100 mg L^−1^ in *M. spicata* “Crispa” was 1.55, phenylalanine 100 mg L^−1^ in *M. piperita* “Granada” was 1.27, phenylalanine 200 mg L^−1^ in *M. piperita* “Granada” was1.22, while in *M. piperita* “Multimentha”, it was 2.00, and tryptophan 100 mg L^−1^ in *M. piperita* “Swiss” was 3.51. Such a result may be related to the fact that phenylalanine and tyrosine are the precursors of the diverse family of phenylpropanoids [31,32]. Phenylalanine and tyrosine are precursors for the diverse group of phenylpropanoids, which include flavonoids, lignins, phenolic acids, stilbenes, and coumarins [33]. The connection between these two amino acids and the synthesis of phenolic compounds was also observed by Manela et al. [34], while increasing the internal concentration of phenylalanine and tyrosine caused a significant increase in phenolic compounds in *Vitis vinifera* cell suspensions. Tryptophan is a precursor for alkaloids, glucosinolates, phytoalexins as well as auxins, while tyrosine for hydroxycinnamic acids, tocopherols, suberin and cyanogenic glycosides and phenylalanine is required for the biosynthesis of simple phenols [33]. 

Phenolic acids are important as compounds with antioxidant, anti-cancer, anti-bacterial and anti-viral properties, finding application in food, pharmaceutics, cosmetics and industries [35,36]. The diverse biological function can lead to their use as natural or derivative pharmaceutical and agricultural chemicals with significant benefits to human health and nutrition [37]. Additionally, they are very important for enhancing the taste, colour, sensory qualities and antioxidant properties in food items and acting as food preservatives. 

Depending on the variety of mint, the total amount of phenolic acids differed 3-fold in the untreated mints. The total amount of phenolic acids was influenced by the variety, as well as treatment, and no similar trends were observed between all varieties of the same species. Despite the fact the phenylalanine is the main precursor of the phenolic acids, an increase in total phenolic acids by 2.1 times was observed only in *M. piperita* “Swiss”, when foliar applied with phenylalanine 100 mg L^−1^. Significantly more effective measures were tryptophane and tyrosine at lower concentrations (100 mg L^−1^). Depending on the variety, as tyrosine is a precursor of the hydroxycinnamic acids, an increase was observed in some phenolic acids under the foliar application with tyrosine. The foliar application of tryptophane at lower concentrations in some mint varieties and at higher concentrations in others promoted the total phenolic acid synthesis from 1.60 to 4.17 times. It may be related to the fact that tryptophane is a precursor of auxin as well as the fact that auxin acts as a morphogen and coordinates plant developmental processes and phototrophy in a concentration-dependent manner. Recent studies suggest that it may directly/indirectly control plant stress [38,39].

Cinnamic and benzoic acids are two groups of phenolic acids. Cinnamic acids include *p*-coumaric, caffeic, ferulic and sinapic acids, while benzoic acids include *p*-hydroxybenzoic, protocatechuic, vanillic, syringic and gallic acids [32]. 

Besides the antioxidation, antimicrobial, antifungal, and antimutagen properties, hydroxybenzoic acid can act as plant growth regulator, and gallic acid may play a protective role in healthy individuals by inhibiting apoptosis [37,40]. The hydroxybenzoic acid synthesis in the mints was influenced by the application of phenylalanine at two concentrations and tryptophane at a lower concentration. In terms of benzoic acid, tyrosine 100 mg L^−1^ was pronounced just in *M. piperita* “Multimentha”.

Much higher amounts of hydroxycinnamic acids are found in mints compared with hydroxybenzoic acids. These phenolic acids have important function in plants adaptation to stress, caused by abiotic and biotic factors [41]. Due to their biological properties and effects in the prevention of various diseases associated with oxidative stress and diverse function for human health hydroxycinnamic acids are very important [42]. *p*-coumaric acid is formed in the phenylpropanoid pathway from phenylalanine with the reaction of phenylalanine ammonia-lyase (PAL) and other enzymes and chemical reactions [33]. According to Barros et al. [43] and Ipson et al. [44], *p*-coumaric acid can also be formed from tyrosine by tyrosine ammonia-lyase (TAL). Cinnamic acid acted as an intermediate in these reactions, the amount of which in the mints was influenced by the variety as well as the application of amino acids. The most effective foliar application was that of phenylalanine and tyrosine. The *p*-coumaric acid undergoes hydroxylation and oxymethylation and produces caffeic and ferulic acids, respectively [45]. The positive correlation between the amounts of cinnamic and *p*-coumaric acids (*r* = 0.488, *p* < 0.05) was established (Table S1). The results of our present study also indicate positive correlations between the amounts of chlorogenic acid and caffeic acid (*r* = 0.674, *p* < 0.05), chlorogenic and ferulic acids (*r* = 0.947, *p* < 0.05) as well as between ferulic and caffeic acid (*r* = 0.693, *p* < 0.05) (Table S1). The amounts of chlorogenic acid in the mints was influenced by all treated amino acids, depending on the variety. The amount of caffeic acid as well *p*-coumaric one in some mint varieties was positively influenced and in some—negatively influenced by tryptophane and phenylalanine, however, it was observed that tyrosine had only a positive effect. Rosmarinic acid is an ester of caffeic acid. Roy and Mukhopadhyay’s [29] investigation showed that tyrosine was very effective for augmenting rosmarinic acid content in *Mentha piperita* L. and nearly increased the production up to 1.77-fold, while phenylalanine significantly affected the production of rosmarinic acid in *Mentha arvensis* L. in in vitro plants. Cinnamic acid content was highly influenced by the foliar application of tyrosine. Water application in some mint varieties also had a positive effect on phenolic acid accumulation.

As seen, foliar application allows for modulating phenols content in mints as potential economically important agricultural plant and to expand the scope of their use and increase the possibilities of application.

## 4. Materials and Methods

### 4.1. Experimental Sites and Soil

This research was conducted in 2017–2018, at the Research Station of Vytautas Magnus University Agriculture Academy in Lithuania. *M. spicata* “Moroccan”, *M. spicata* “Crispa”, *M. piperita* “Granada”, *M. piperita* “Swiss”, and *M. piperita* “Multimentha” were planted on 4–5 May 2017, field location 54°53′8.9″ N, 23°50′8.02″ E. The soil at the experimental site was silty loam (46% sand, 42% silt, and 12% clay) Endohypogleyic-Eutric Planosol (Ple-gln-w) (IUSS Working Group WRB, 2014). Split-plot was used in the experimental design in four replications and the plot area was 800 m^2^ consisting of five rows (4 m in length and 50 cm between rows). The arable soil layer pH was between 7.20 and 7.70, mineral nitrogen from 5.75 to 7.24, phosphorus from 249 to 260 and potassium from 141 to 130 mg kg^−1^ of soil. The mint plants were sprayed with aromatic amino acids L-phenylalanine, L-tryptophan and L-tyrosine at two concentrations: 100 mg L^−1^ and 200 mg L^−1^ three times with a 15-day interval. The mints were sprayed with amino acids until the solution started dripping from plants. Tween 20 was added to spraying solution as a surfactant. The first spraying was started at a mint development stage BBCH 21. Foliar spray with water was used as a positive control. The effects of all treatments were compared with a negative control (without spraying). The plants were harvested 15 days after the last spray at mint development stage BBCH 65 [46]. Mints leaves were collected in bags and stored until the freeze drying at −34 °C.

### 4.2. Meteorological Conditions

**Figure 5:**
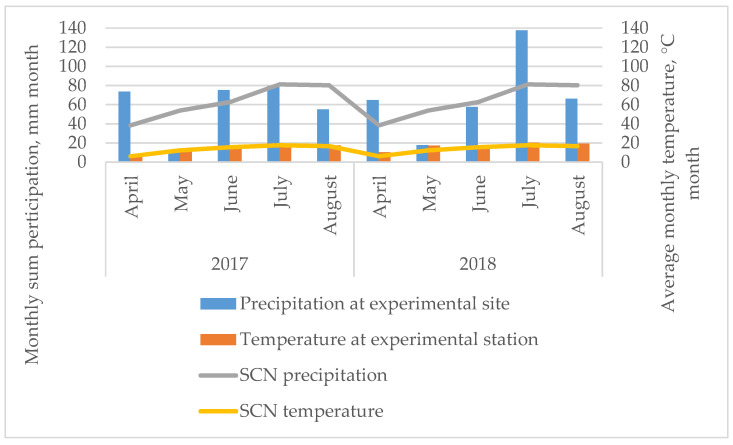
Monthly mean precipitation and temperature at the experimental sites. SCN—standard climate norm, average data for 39 years (1974–2013).

The meteorological conditions during the vegetative period of mints are presented in Figure 5.

According to the weather data, the amount of precipitation was distributed very unevenly in 2017 and 2018. In 2017, the precipitation at the beginning of the vegetation period at the experimental site (in April) was higher than the long-term average of 1974–2013, while the precipitation in May and August was 5.12- and 1.46-times lower than the standard climate normal. The same trends were observed in 2018 when, in May and August, the precipitation was 3.06- and 1.21-times lower than SCN. During the experiment in 2018, May was extremely dry, as in 2017. The highest precipitation during the experiment was observed in April 2017 and 2018—by 1.92 and 1.69 times, respectively, and by 1.69 times in 2018, compared with the standard climate normal. The air temperature of the 2017 and 2018 growing seasons differed slightly from the long-term average, except for April and May 2018, when the monthly average temperature was 1.67- and 1.40-times higher than the standard climate normal. The meteorological conditions were very variable in both years and plants grew under stress conditions.

### 4.3. Chemicals

Methanol, acetonitrile, external standards, such as gallic acid, chlorogenic acid, *β*- coumaric acid, with a purity of 99.5%, were purchased from Sigma-Aldrich and Fluka (Warsaw, Poland). Tween 20, L-tryptophan, L-phenylalanine, L-tyrosine were purchased from Carl Roth (Karlsruhe, Germany).

### 4.4. Methods of Sample Preparation

After harvest, mint leaves were freeze dried by SCANVAC Coolsafe 55-9 lyophilizer for 24 h at −60 °C and finally ground to a fine powder in a laboratory mill (Grindomix GM 200, Retsh GmbH, Haan, Germany). For polyphenol content analysis, a total of 100–200 g samples were taken from each replicate. Prior to analyses, the samples were stored at −80 °C.

### 4.5. Polyphenol Content

A total of 100 mg of freeze-dried mint powder was mixed with 5 mL of 80% methanol in a plastic test tube, then mixed thoroughly by vortex and incubated in an ultrasonic bath for 15 min at 30 °C. The samples were centrifuged at 5000 rpm. Then, 1 mL of extract was collected from the test tube and re-centrifuged at 12,000 rpm. The amount of 500 μL of extract was taken for HPLC vials and analysed. A Synergi Fusion-RP 80i Phenomenex column (250 × 4.60 mm) was used for the analysis of phenolic compounds. Shimadzu equipment (two pumps (LC-20AD), a controller (CBM-20A), a column oven (SIL-20AC), and UV-vis spectrometer (SPD-20 AV)) was used to carry out the analysis. The gradient flow was applied along with two mobile phases—10% (*v*/*v*) acetonitrile and ultrapure water (solvent A) and 55% (*v*/*v*) acetonitrile and ultrapure water (solvent B), pH 3; the used gradient program was: 0–21 min, 95% solvent A and 5% solvent B; 22–25 min, 50% solvent A and 50% solvent B; 26–27 min, 20% solvent A and 80% solvent B; 28–32 min, 20% solvent A and 80% solvent B; 32–36 min, 95% solvent A and 5% solvent B. The analysis duration 36 min, flow 1 mL min^−1^, wavelength for flavonoids was 250 nm, for the phenolic acids −370 nm. Retention times for identified phenolic acids are presented in Table S2. The total amount of polyphenols is a sum of total flavonoids and total phenolic acids [47].

### 4.6. Statistical Analysis

All analyses were performed in triplicate. The data analysis was conducted with STATISTICA version 12 software (StatSoft, Inc., Tulsa, OK, USA). The interaction between years and phenolic compound content was calculated using two-way analysis of variance (ANOVA). The Fisher’s test was applied to assess significant differences (*p* < 0.05) between samples. Then, the results were analysed using one-way analysis of variance (ANOVA). The Tukey’s Honestly significant difference test (HSD) was applied to assess significant differences between mean values (*p* < 0.05). The relationship between the values was determined using the Pearson’s linear correlation coefficient (*p* < 0.05). The principal component analysis (PCA) was performed to evaluate the relationships between the applications of the amino acids, mint variety and chemical content, as well as the hierarchical cluster analysis (HCA), which was performed to categorize the mints based on their phenolic compound content with XLSTAT software version 2019.3.02 (Addinsoft, Paris, France).

## 5. Conclusions

Phenolic compounds perform various functions in plants, as well as affecting various systems of the human body, are important in various fields of industry, so promoting their synthesis in mints, an economically important plant, not only in vitro but also under the field conditions, is meaningful. Spraying with amino acids can increase the amount of total phenolic compounds and total phenolic acids, as well as influence the synthesis of different phenolic acids. The combination of mint variety and the foliar application of amino acids can be pronounced for the synthesis of phenols. Of great interest is the effect of amino acids on phenols accumulation in *Mentha piperita* “Swiss”. This mint variety has the highest number of total phenols, as well as phenolic acids, and the most intensive response to the foliar application of amino acids. In *Mentha piperita* “Swiss” and *Mentha piperita* “Multimentha” did not decrease the amount of benzoic acid after foliar application as in other mints varieties. However, further research and investigations are essential to establish the possibilities to model the composition of agricultural commodities in terms of aromatic plants.

## Figures and Tables

**Table 1:** Gallic acid content in mints influenced by foliar application of amino acids, mg 100 g^−1^ DM in 2017–2018.

Treatment	Species/Variety				
	*M. spicata* “Moroccan”	*M. spicata* “Crispa”	*M. piperita* “Granada”	*M. piperita* “Swiss”	*M. piperita* “Multimentha”
Water	6.82 * ± 0.34 abcBC	7.60 ± 0.52 cC	6.08 ± 0.18 aC	4.75 ± 0.16 aA	11.21 ± 0.53 dD
Non-sprayed	6.57 ± 0.08 abcA	7.23 ± 0.42 cA	6.34 ± 0.25 aA	22.02 ± 2.84 cC	13.69 ± 0.69 eB
Phenylalanine 100 mg L^−1^	7.12 ± 0.08 cB	8.95 ± 0.53 dA	8.66 ± 0.84 bA	8.40 ± 0.27 abA	6.39 ± 0.07 abB
Phenylalanine 200 mg L^−1^	7.16 ± 0.30 cA	7.58 ± 0.09 cA	13.84 ± 0.42 cC	5.91 ± 0.23 abB	7.62 ± 0.31 bA
Tryptophan 100 mg L^−1^	6.79 ± 0.27 abcA	5.98 ± 0.16 bA	6.82 ± 0.18 aA	24.81 ± 1.39 cC	16.70 ± 1.29 fB
Tryptophan 200 mg L^−1^	6.22 ± 0.33 aB	4.94 ± 0.10 aA	8.06 ± 0.18 bC	23.07 ± 0.73 cD	5.32 ± 0.29 aAB
Tyrosine 100 mg L^−1^	6.98 ± 0.08 bcC	7.31 ± 0.24 cC	5.97 ± 0.13 aB	4.11 ± 0.30 aA	9.46 ± 0.37 cD
Tyrosine 200 mg L^−1^	6.45 ± 0.17 abB	6.96 ± 0.13 cB	6.82 ± 0.31 aB	4.74 ± 0.53 aA	13.19 ± 0.32 eC

* Means ± standard deviations marked with different upper letters (A, B, C…) in the rows are significant different at *p* < 0.05; Means ± standard deviations marked with different lower letters (a, b, c…) in the columns are significant different at *p* < 0.05.

**Table 2:** Benzoic acid content in mints influenced by foliar application of amino acids, mg 100 g^−1^ DM in 2017–2018.

Treatment	Species/Variety				
	*M. spicata* “Moroccan”	*M. spicata* “Crispa”	*M. piperita* “Granada”	*M. piperita* “Swiss”	*M. piperita* “Multimentha”
Water	2.03 * ± 0.38 abAB	2.96 ± 0.41 bB	5.36 ± 0.39 dC	1.91 ± 0.38 abA	4.96 ± 0.43 cdC
Non-sprayed	4.30 ± 0.39 eBC	5.13 ± 1.07 cC	2.44 ± 0.38 bcAB	0.92 ± 0.31 aA	3.42 ± 1.59 bcBC
Phenylalanine 100 mg L^−1^	3.02 ± 0.38 bcB	0.89 ± 0.37 aA	2.91 ± 0.41 cB	1.12 ± 0.37 aA	0.93 ± 0.33 aA
Phenylalanine 200 mg L^−1^	1.62 ± 0.41aA	5.35 ± 0.40 cBC	1.92 ± 0.31 bA	5.24 ± 0.40 eBC	7.17 ± 0.41 deC
Tryptophan 100 mg L^−1^	3.13 ± 0.38 cdAB	2.38 ± 0.44 bAB	2.17 ± 0.29 bcA	3.42 ± 0.44 cdB	8.90 ± 0.78 eC
Tryptophan 200 mg L^−1^	1.99 ± 0.38 abA	2.04 ± 0.39 abA	2.10 ± 0.40 bcA	2.28 ± 0.40 bA	1.46 ± 0.39 abA
Tyrosine 100 mg L^−1^	4.13 ± 0.39 deB	2.05 ± 0.38 abA	0.53 ± 0.37 aA	4.34 ± 0.55 deB	7.82 ± 1.36 eC
Tyrosine 200 mg L^−1^	3.03 ± 0.38 bcC	2.23 ± 0.38 abBC	1.929 ± 0.35 bcB	2.58 ± 0.38 bcBC	0.92 ± 0.44 aA

* Means ± standard deviations marked with different upper letters (A, B, C…) in the rows are significant different at *p* < 0.05; Means ± standard deviations marked with different lower letters (a, b, c…) in the columns are significant different at *p* < 0.05.

**Table 3:** Chlorogenic acid content in mints influenced by foliar application of amino acids, mg 100 g^−1^ DM in 2017–2018.

Treatment	Species/Variety				
	*M. spicata* “Moroccan”	*M. spicata* “Crispa”	*M. piperita* “Granada”	*M. piperita* “Swiss”	*M. piperita* “Multimentha”
Water	11.15 * ± 2.68 aA	2.14 ± 0.04 aA	7.61 ± 0.19 abA	130.27 ± 14.23 bB	6.13 ± 0.30 abA
Non-sprayed	14.93 ± 0.36 bcA	2.09 ± 0.05 aA	7.09 ± 0.18 abA	48.28 ± 14.63 aB	8.25 ± 2.64 abA
Phenylalanine 100 mg L^−1^	16.29 ± 1.50 bcC	2.02 ± 0.10 aA	10.43 ± 0.59 cB	123.63 ± 4.57 bD	9.54 ± 0.65 bcB
Phenylalanine 200 mg L^−1^	15.75 ± 0.59 bcAB	2.35 ± 0.16 aA	12.47 ± 0.30 dA	67.92 ± 12.19 aC	28.10 ± 5.57 dB
Tryptophan 100 mg L^−1^	16.79 ± 0.56 cB	1.93 ± 0.05 aA	6.89 ± 0.43 aAB	114.43 ± 12.45 bC	15.49 ± 3.05 cAB
Tryptophan 200 mg L^−1^	13.46 ± 0.33 abA	3.55 ± 0.09 aA	11.35 ± 0.20 cdA	103.68 ± 13.04 bB	9.63 ± 0.76 bcA
Tyrosine 100 mg L^−1^	15.40 ± 0.42 bcB	12.54 ± 0.33 bB	19.42 ± 1.09 eC	73.86 ± 2.44 aD	3.87 ± 1.69 abA
Tyrosine 200 mg L^−1^	14.93 ± 0.37 bcB	14.71 ± 1.74 cB	8.33 ± 0.23 bAB	64.38 ± 5.65 aC	2.51 ± 0.80 aA

* Means ± standard deviations marked with different upper letters (A, B, C…) in the rows are significant different at *p* < 0.05; Means ± standard deviations marked with different lower letters (a, b, c…) in the columns are significant different at *p* < 0.05.

**Table 4:** Caffeic acid content in mints influenced by foliar application of amino acids, mg 100 g^−1^ DM in 2017–2018.

Treatment	Species/Variety				
	*M. spicata* “Moroccan”	*M. spicata* “Crispa”	*M. piperita* “Granada”	*M. piperita* “Swiss”	*M. piperita* “Multimentha”
Water	53.34 * ± 12.04 aB	9.26 ± 1.15 aA	46.24 ± 7.51 bcB	90.55 ± 15.44 aC	9.21 ± 1.75 abA
Non-sprayed	55.93 ± 9.80 aB	7.64 ± 1.17 aA	47.87 ± 8.37 bcB	22.59 ± 13.64 aA	7.50 ± 0.94 abA
Phenylalanine 100 mg L^−1^	77.97 ± 15.72 aA	11.39 ± 1.86 aB	56.55 ± 15.98 cA	58.79 ± 8.24 aA	5.18 ± 0.56 aB
Phenylalanine 200 mg L^−1^	83.01 ± 14.13 aC	6.41 ± 0.89 aA	46.81 ± 8.79 bcB	55.47 ± 9.51 aB	6.88 ± 1.33 aA
Tryptophan 100 mg L^−1^	82.66 ± 13.43 aB	4.65 ± 0.52 aA	19.82 ± 3.30 aAB	340.87 ± 61.93 bC	7.14 ± 0.82 abA
Tryptophan 200 mg L^−1^	62.41 ± 10.96 aA	5.25 ± 0.68 aA	20.02 ± 3.34 aA	300.32 ± 53.78 bB	12.02 ± 4.29 bA
Tyrosine 100 mg L^−1^	82.56 ± 17.22 aB	40.56 ± 7.08 bAB	25.17 ± 9.82 abAB	338.18 ± 60.58 bC	7.87 ± 1.64 abA
Tyrosine 200 mg L^−1^	65.76 ± 11.57 aB	6.56 ± 1.12 aA	48.38 ± 7.02 bcAB	277.64 ± 44.96 bC	6.05 ± 0.83 aA

* Means ± standard deviations marked with different upper letters (A, B, C…) in the rows are significant different at *p* < 0.05; Means ± standard deviations marked with different lower letters (a, b, c…) in the columns are significant different at *p* < 0.05.

**Table 5:** *p*-coumaric acid content in mints influenced by foliar application of amino acids, mg 100 g^−1^ DM in 2017–2018.

Treatment	Species/Variety				
	*M. spicata* “Moroccan”	*M. spicata* “Crispa”	*M. piperita* “Granada”	*M. piperita* “Swiss”	*M. piperita* “Multimentha”
Water	104.45 * ± 8.45 bC	28.41 ± 4.57 abA	58.53 ± 5.38 abB	65.54 ± 5.72 dB	48.30 ± 10.22 bB
Non-sprayed	117.27 ± 11.05 bB	33.61 ± 2.34 bA	105.84 ± 9.91 cdB	38.10 ± 3.07 bA	30.16 ± 1.95 aA
Phenylalanine 100 mg L^−1^	120.79 ± 11.62 bB	48.89 ± 4.83 cdA	134.99 ± 14.99 dB	28.85 ± 1.90 bA	28.51 ± 1.57 aA
Phenylalanine 200 mg L^−1^	65.66 ± 5.85 aC	37.68 ± 3.49 bcB	134.87 ± 18.28 dD	15.14 ± 0.76 aA	28.26 ± 1.79 aAB
Tryptophan 100 mg L^−1^	120.10 ± 11.38 bC	76.82 ± 8.98 eB	53.21 ± 5.95 abA	64.47 ± 6.38 dAB	48.84 ± 4.16 bA
Tryptophan 200 mg L^−1^	97.72 ± 9.08 bC	31.34 ± 2.40 bA	51.37 ± 3.62 abB	29.47 ± 3.26 bA	55.55 ± 5.27 bB
Tyrosine 100 mg L^−1^	111.65 ± 12.67 bC	60.01 ± 5.71 dB	44.22 ± 3.04 aAB	28.54 ± 1.93 bA	30.07 ± 5.38 aA
Tyrosine 200 mg L^−1^	98.57 ± 9.16 bD	16.22 ± 1.62 aA	76.85 ± 15.51 bcC	51.71 ± 3.75 cB	52.65 ± 3.93 bB

* Means ± standard deviations marked with different upper letters (A, B, C…) in the rows are significant different at *p* < 0.05; Means ± standard deviations marked with different lower letters (a, b, c…) in the columns are significant different at *p* < 0.05.

**Table 6:** Ferulic acid content in mints influenced by foliar application of amino acids, mg 100 g^−1^ DM in 2017–2018.

Treatment	Species/Variety				
	*M. spicata* “Moroccan”	*M. spicata* “Crispa”	*M. piperita* “Granada”	*M. piperita* “Swiss”	*M. piperita* “Multimentha”
Water	17.34 * ± 1.65 bcdB	2.36 ± 0.18 aA	5.19 ± 0.37 bA	176.13 ± 9.57 dC	4.18 ± 0.32 abA
Non-sprayed	19.26 ± 0.99 cdeC	3.16 ± 0.27 aA	22.79 ± 1.06 aD	27.10 ± 1.41 aE	7.22 ± 0.50 cB
Phenylalanine 100 mg L^−1^	20.59 ± 1.19 dcA	2.64 ± 0.24 aB	24.07 ± 1.61 aD	113.38 ± 12.62 bcC	30.79 ± 1.91 eA
Phenylalanine 200 mg L^−1^	7.34 ± 0.42 aAB	3.53 ± 0.28 aA	20.42 ± 1.51 aCD	98.17 ± 11.90 bcC	2.98 ± 0.34 aA
Tryptophan 100 mg L^−1^	22.11 ± 2.17 eB	2.67 ± 0.29 aA	18.12 ± 1.66 cdBC	115.80 ± 13.14 cC	2.29 ± 0.19 aA
Tryptophan 200 mg L^−1^	15.64 ± 0.93 bB	2.41 ± 0.22 aA	16.28 ± 0.98 cB	112.15 ± 6.18 bcD	23.22 ± 1.00 dC
Tyrosine 100 mg L^−1^	18.08 ± 1.21 bcdA	7.82 ± 0.51 bA	47.98 ± 2.12 eE	116.21 ± 12.16 cC	7.04 ± 0.47 cA
Tyrosine 200 mg L^−1^	17.04 ± 1.01 bcB	15.76 ± 1.14 cB	7.34 ± 0.49 bA	88.43 ± 4.17 bC	6.12 ± 0.77 bcA

* Means ± standard deviations marked with different upper letters (A, B, C…) in the rows are significant different at *p* < 0.05; Means ± standard deviations marked with different lower letters (a, b, c…) in the columns are significant different at *p* < 0.05.

**Table 7:** Cinnamic acid content in mints influenced by foliar application of amino acids, mg 100 g^−1^ DM in 2017–2018.

Treatment	Species/Variety				
	*M. spicata* “Moroccan”	*M. spicata* “Crispa”	*M. piperita* “Granada”	*M. piperita* “Swiss”	*M. piperita* “Multimentha”
Water	14.86 * ± 1.73 cD	0.30 ± 0.05 aA	4.80 ± 0.21 aB	7.18 ± 0.39 cC	1.03 ± 0.96 aA
Non-sprayed	13.97 ± 0.81 cB	0.34 ± 0.03 aA	20.74 ± 1.23 bC	0.23 ± 0.03 aA	1.00 ± 0.92 aA
Phenylalanine 100 mg L^−1^	14.88 ± 1.11 cC	0.19 ± 0.03 aA	4.44 ± 0.25 aB	0.45 ± 0.02 aA	1.00 ± 0.93 aA
Phenylalanine 200 mg L^−1^	1.23 ± 0.08 aA	0.16 ± 0.04 aA	21.64 ± 1.28 bC	3.51± 0.19 bB	0.07 ± 0.05 aA
Tryptophan 100 mg L^−1^	15.39 ± 1.26 cB	0.07 ± 0.04 aA	23.40 ± 1.80 bC	0.23 ± 0.06 aA	1.15 ± 0.09 aA
Tryptophan 200 mg L^−1^	11.08 ± 0.65 bB	0.08 ± 0.04 aA	22.32 ± 1.41 bC	0.63 ± 0.98 aA	0.48 ± 0.39 aA
Tyrosine 100 mg L^−1^	14.05 ± 1.17 cD	11.22 ± 0.64 cC	22.55 ± 1.60 bE	4.48 ± 0.21 bB	0.48 ± 0.46 aA
Tyrosine 200 mg L^−1^	10.64 ± 0.61 bD	1.14 ± 0.08 bB	7.27 ± 0.40 aC	0.18 ± 0.05 aA	0.26 ± 0.03 aA

* Means ± standard deviations marked with different upper letters (A, B, C…) in the rows are significant different at *p* < 0.05; Means ± standard deviations marked with different lower letters (a, b, c…) in the columns are significant different at *p* < 0.05.

## Data Availability

Not applicable.

## Supplementary Materials

The following are available online at https://www.mdpi.com/2223-7747/10/3/599/s1, Table S1: Correlation between separate phenolic acids content in mints, 2017–2018, Table S2: Retention time for identified phenolic acids in mints leaves (based on standards solutions).
