# Supplementary material for: Impact of Foliar Application of Amino Acids on Total Phenols, Phenolic Acids Content of Different Mints Varieties under the Field Condition
Source: Plants (Basel). 2021 Mar 23;10(3):599. doi: 10.3390/plants10030599 (PMC8005027; doi:10.3390/plants10030599)
Supplement: Supplementary file 1 [file plants-10-00599-s001.pdf]

plants

MDPI

Supplementary Materials

Impact of Foliar Application of Amino Acids on Total Phenols, Phenolic Acids Content of Different Mints Varieties under the Field Condition

Živilė Tarasevičienė *, Aloyzas Velička and Aurelija Palauskienė

Institute of Agricultural and Food Sciences, Faculty of Agronomy, Vytautas Magnus University Agriculture Academy, 53361 Akademija, Lithuania; aloyzas.velicka@vdu.lt (A. V.); aurelija.paulauskiene@vdu.lt (A. P.)
* Correspondence: zivile.tarasevicienе@vdu.lt; Tel.: +370 61151028

Table S1. Correlation between separate phenolic acids content in mints, 2017–2018.

Phenolic Acids	Gallic	Chloroge-nic	Caffeic	*p*-coumaric	Ferulic	Benzoic	Cinamic
Gallic	-	0.327	n.s.	n.s.	n.s.	n.s.	n.s.
Chlorogenic	0.328*	-	0.673	n.s.	0.947	n.s.	n.s.
Caffeic acid	n.s.	0.674	-	n.s.	0.693	n.s.	n.s.
p-coumaric	n.s.	n.s.	n.s.	-	n.s.	n.s.	0.488
Ferulic	n.s.	0.947	0.692	n.s.	-	n.s.	n.s.
Benzoic	n.s.	n.s.	n.s.	n.s.	n.s.	-	n.s.
Cinamic	n.s.	n.s.	n.s.	0.488	n.s.	n.s.	-

*$p < 0.05$.

Table S2. Retention time for identified phenolic acids in mints leaves (based on standards solutions)

Identified Compounds	Retention Time (min)
Gallic acid	4.62
Chlorogenic acid	11.72
Caffeic acid	14.83
p-coumaric acid	20.10
Ferulic acid	21.51
Benzoic acid	24.96
Cinamic acid	25.22
